# Effect of intramolecular hydrogen-bond formation on the molecular conformation of amino acids

**DOI:** 10.1038/s42004-020-0329-7

**Published:** 2020-06-30

**Authors:** Giulia Giubertoni, Oleksandr O. Sofronov, Huib J. Bakker

**Affiliations:** AMOLF, Science Park 104, 1098 XG Amsterdam, The Netherlands

**Keywords:** Biochemistry, Physical chemistry

## Abstract

The molecular conformation of the carboxyl group can be crucial for its chemical properties and intermolecular interactions, especially in complex molecular environments such as polypeptides. Here, we study the conformational behaviour of the model amino acid N-acetylproline in solution at room temperature with two-dimensional infrared spectroscopy. We find that the carboxyl group of N-acetylproline adopts two distinct conformations, *syn-* and *anti-*. In the *syn-*conformer the O–H group is oriented at  ~60^∘^ with respect to the C=O and in the *anti-*conformer the O–H is anti-parallel to the C=O. In hydrogen-bond accepting solvents such as dimethyl sulfoxide or water, we observe that, similar to simple carboxylic acids, around 20% of the -COOH groups adopt an *anti-*conformation. However, when N-acetylproline is dissolved in a weakly hydrogen-bond accepting solvent (acetonitrile), we observe the formation of a strong intramolecular hydrogen bond between the carboxyl group in the *anti-*conformation and the amide group, which stabilizes the *anti-*conformer, increasing its relative abundance to ~60%.

## Abstract (web summary)

The properties of carboxyl compounds depend on their conformations, but characterising each conformation can be challenging. Here, femtosecond spectroscopy reveals that the carboxyl group of N-acetylproline preferentially adopts a *syn* conformation in water, but in apolar environments an intramolecular hydrogen favours an *anti* conformation.

## Introduction

Amino acids fulfill diverse roles in living systems as protein building blocks, neurotransmitters, and metabolic intermediates, thereby making them one of the most important classes of organic molecules^1–3^. In view of its low pK_*a*_ values of  ~2 in aqueous solution, the carboxyl group of the amino acid will usually be deprotonated. However, under specific conditions, like the nonpolar microenvironments of polypeptides, the side-chain carboxyl groups of aspartic and glutamic acids, and the carboxyl groups of C-terminal amino acids can exist in their protonated form^4–6^, and can participate in enzymatic processes or in the stabilization of protein tertiary structure^7,8^. Recently, we showed that the carboxyl group of simple carboxylic acids in room temperature solution adopts two distinct nearly planar conformations, syn- (70–80% fraction) and anti- (20–30%)^9^. In the syn-conformation the hydroxyl group is oriented at an angle of 60° with respect to the carbonyl, and in the anti-conformation the hydroxyl group is oriented anti-parallel with respect to the carbonyl group^10,11^. It has also been shown that the reactivity of the carboxyl group, and more general the strength of the intra- and intermolecular interactions, strongly depends on the conformation of the carboxyl group^12,13^.

**Fig. 1:**
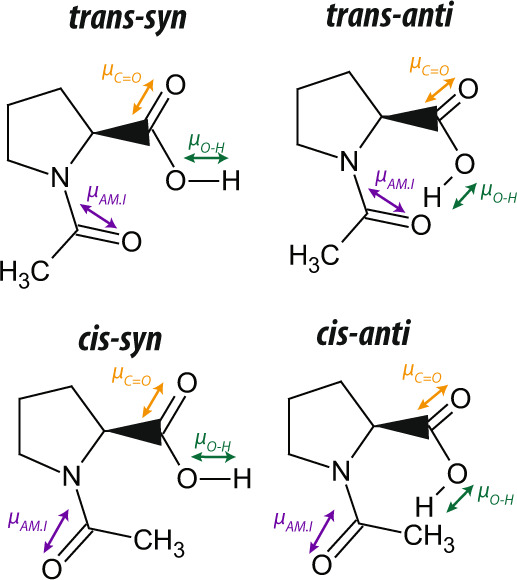
Conformational isomers of *N*-acetylproline. Schematic illustration of the different conformational isomers of *N*-acetylproline as determined by the conformations of the amide and carboxyl groups. Green and yellow arrows indicate the orientation of the transition dipole moments of the hydroxyl and carbonyl stretch vibrations. The purple arrow indicates the transition dipole moment of the amide I vibration.

Here we study the conformational isomerism of the carboxyl group of the model acetylated amino acid *N*-acetylproline in weakly and strongly interacting solvents with polarization-resolved two-dimensional infrared spectroscopy (2DIR). Figure 1 shows all the possible structures of *N*-acetylproline considering the conformational isomerism of both the amide (cis/trans) and the carboxyl group (syn/anti). We here do not consider the possible conformational isomers due to the rotation of the carboxyl group around the C–C bond since our experiments are not sensitive to this rotation. In Fig. 1, it is seen that the trans-anti conformation offers the possibility of forming an intramolecular hydrogen bond between the C=O group of the amide group and the O–H group of the carboxyl group. Hence, *N*-acetylproline represents an ideal system to study the competition of inter- and intramolecular interactions on the molecular conformation in solvents that resemble hydrophobic and hydrophilic environments. The amide and hydroxyl vibration are usually not as strongly coupled as the carboxyl and hydroxyl vibrations since they are a few atoms apart. Nevertheless, in the presence of an intra-molecular hydrogen-bond, the two molecular groups are interacting, and, thus, the two molecular vibrations can be coupled leading to the observation of cross-peak signatures.

## Results

### Linear infrared spectra

**Fig. 2:**
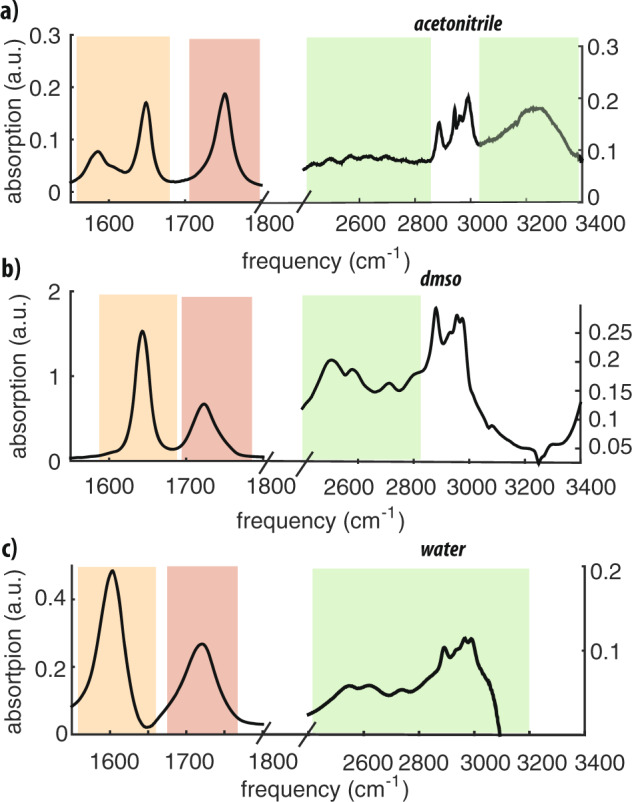
Molecular vibrations of carboxyl and amide groups. Fourier transform infrared spectra of *N*-acetylproline in **a** acetonitrile (0.08 M), **b** DMSO (0.4 M), and **c** water (1 M). The solvent background is subtracted. The orange, red, and green shaded areas indicate the frequency regions of the amide I, the carbonyl, and the hydroxyl vibrations, respectively.

Figure 2 shows the linear infrared spectra of *N*-acetylproline dissolved in acetonitrile, dimethyl sulfoxide (DMSO), and water in the frequency regions of the amide I, carboxyl C=O stretch, and carboxyl O–H stretch vibrations. We observe that the maximum frequency of the carboxyl C=O stretch absorption shifts from 1752 cm^−1^ in acetonitrile to ~1720 cm^−1^ in DMSO and water. The same trend is observed for the amide I vibration, which shifts from 1648 cm^−1^ in acetonitrile to 1643 cm^−1^ in DMSO and to  ~1600 cm^−1^ in water. When *N*-acetylproline is dissolved in acetonitrile, two additional amide peaks appear at 1585 cm^−1^ and 1605 cm^−1^. The absorption band at 1605 cm^−1^ is found to be concentration dependent and can be assigned to the amide I vibration of the *N*-acetylproline dimer (see Supplementary Discussion and Supplementary Fig. 1).

The absorption spectrum of the carboxyl O–H stretch vibrations is also strongly solvent dependent. When *N*-acetylproline is dissolved in acetonitrile, the carboxyl O–H stretch spectrum extends to frequencies up to 3400 cm^−1^. The observed broad absorption spectrum can be subdivided into a broad continuous absorption below 2900 cm^−1^ and a broad band centered ~3250 cm^−1^. When *N*-acetylproline is dissolved in DMSO or water, a strong absorption is observed in the frequency region 2400–2900 cm^−1^ due to strongly hydrogen-bonded acidic O–H groups. For *N*-acetylproline dissolved in water, the spectrum at frequencies  >3000 cm^−1^ is not accessible due to strong absorption of water molecules. Due to coupling of the O–H stretch vibrations to low-frequency hydrogen bond modes, the absorption spectrum contains a series of subbands^14,15^.

### Conformers of *N*-acetylproline in different solvents

**Fig. 3:**
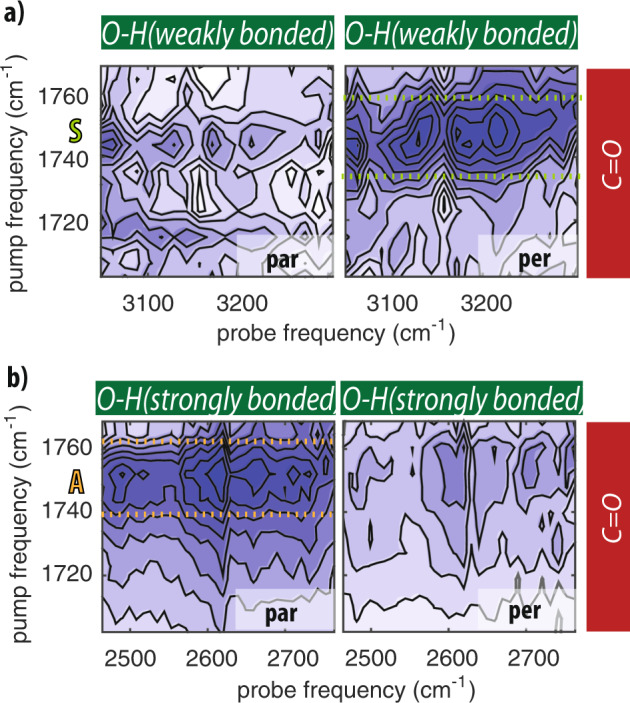
Syn- and anti-conformers in hydrophobic environment. 2DIR spectra of *N*-acetylproline dissolved in acetonitrile measured by exciting the carboxyl C=O vibration and probing **a** high frequency and **b** low-frequency carboxyl O–H vibrations. The spectra are measured in parallel polarization configuration (left panel) and perpendicular polarization configuration (right panel). The concentration of *N*-acetylproline is 0.2 M (**a**) and 0.08 M (**b**). The spectra are measured at a time delay of 0.5 ps. The color scale is normalized to the maximum intensity of the perpendicular signal in **a**, and to that of the parallel signal in **b**.

Figure 3 shows 2DIR measurements for *N*-acetylproline dissolved in acetonitrile, where we excite the carbonyl modes, and we probe the hydroxyl modes. The coupling between the carbonyl and the hydroxyl vibrations results in a negative absorption change (bleach). The cross-peak intensity is strongly dependent on the polarization. Figure 3a shows the cross-peak signal obtained by exciting the carbonyl near 1750 cm^−1^, and probing the O–H stretch vibration near 3200 cm^−1^. The cross-peak signals have their maximum at an excitation frequency 1748 cm^−1^, and a broad frequency range of the O–H stretch vibration. By extracting the cross-peak intensity measured in parallel (Δ*α*_par_) and perpendicular (Δ*α*_per_) polarization configuration, we determine the angle between the transition dipoles moments of the carbonyl and hydroxyl vibrations (see Supplementary Discussion). We thus find an angle of 75 ± 10°. This angle is in good agreement with the angle that we have found before for the carboxyl group of formic acid in syn-conformation^9^. We thus conclude that at an excitation frequency of 1748 cm^−1^, mainly carbonyl groups of syn-conformers absorb. Figure 3b shows the 2DIR spectrum obtained when exciting the carbonyl vibrations and probing the low-frequency hydroxyl vibrations ~2600 cm^−1^. We observe that the largest cross-peak signal is obtained in parallel polarization configuration with a maximum at an excitation frequency of 1755 cm^−1^. From the signals measured in parallel and perpendicular polarization configuration we find that the angle between the carbonyl and the hydroxyl transition dipole moments amounts to 17 ± 10°. This value is in good agreement with that of a carboxyl group in anti-conformation^9^.

**Fig. 4:**
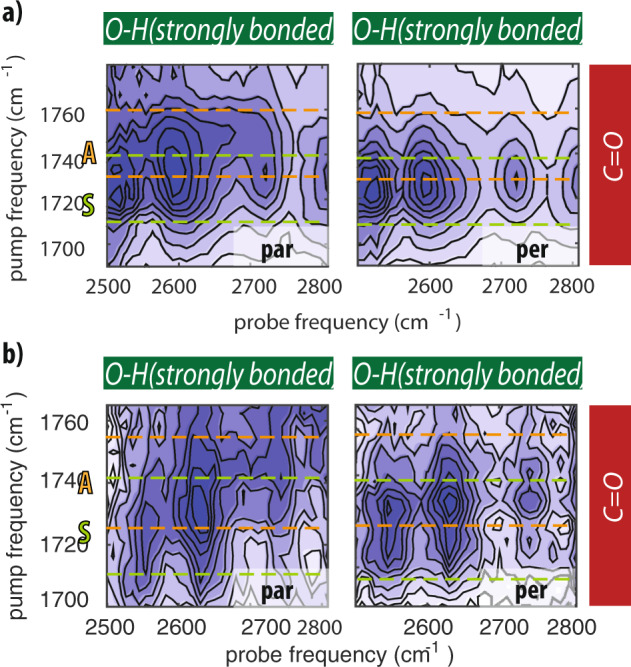
Syn- and anti-conformers in hydrophilic environment. 2DIR spectra in the C=O/O–H cross-peak region measured in parallel polarization configuration (left panel) and perpendicular polarization configuration (right panel), for solutions of *N*-acetylproline in DMSO (**a**) and in water (**b**) at a time delay of 0.5 and 0.3 ps, respectively.

In Fig. 4 we show C=O/O–H cross-peak 2DIR spectra of *N*-acetylproline dissolved in DMSO and in water, measured in parallel and perpendicular polarization configurations. The excitation of the high-frequency carbonyl vibrations near 1745 cm^−1^ results in a stronger response of the O–H stretch vibrations in parallel polarization configuration, while excitation of the low-frequency carbonyl vibrations near 1730 cm^−1^ leads to a stronger cross-peak signal of the O–H vibrations in perpendicular polarization configuration. Hence, in DMSO and in aqueous solution the O–H stretch vibrations of both the syn- and the anti- conformer of the *N*-acetylproline carboxyl group absorb in the same broad frequency range (2400–2900 cm^−1^), and we observe a significant difference in the carbonyl stretch frequency between the two conformers.

The large difference between the frequencies of the O–H stretch vibrations of the syn- and anti-conformers observed for *N*-acetylproline dissolved in acetonitrile indicates a large difference in the strength of the hydrogen bond donated by the O–H group. The O–H stretch vibration has a high frequency of  ~3200 cm^−1^ when the carboxyl group has a syn-conformation, and decreases to  ~2600 cm^−1^ when carboxyl group acquires an anti-conformation. This finding indicates that the O–H group of the anti-conformer forms a strong intramolecular hydrogen bond within the *N*-acetylproline molecule, while the O–H group of the syn-conformers is only weakly hydrogen bonded to the solvent. This notion is supported by the relatively low vibrational frequency of the C=O vibration of the anti-conformer in acetonitrile. In previous work we observed that the carbonyl vibration of the anti-conformer of formic acid in acetonitrile solution absorbs at a frequency that is 20–30 cm^−1^ higher than the carbonyl vibration of the syn-conformer^9^. However, the frequency of the carbonyl vibration can decrease by 20–30 cm^−1^ upon strong hydrogen bonding of the O–H group. We clearly see this effect when we compare the C=O vibrational spectrum of *N*-acetylproline in acetonitrile and DMSO solutions (Fig. 2). For *N*-acetylproline dissolved in acetonitrile the frequency difference between the carbonyl vibrations of the anti-conformer and syn-conformer is much smaller than for *N*-acetylproline in DMSO or water, because the usual blueshift of the carbonyl vibration of the anti-conformer in comparison with the syn-conformer gets largely compensated by the redshift that results from the strong intramolecular hydrogen bond formed by the anti-conformer.

**Fig. 5:**
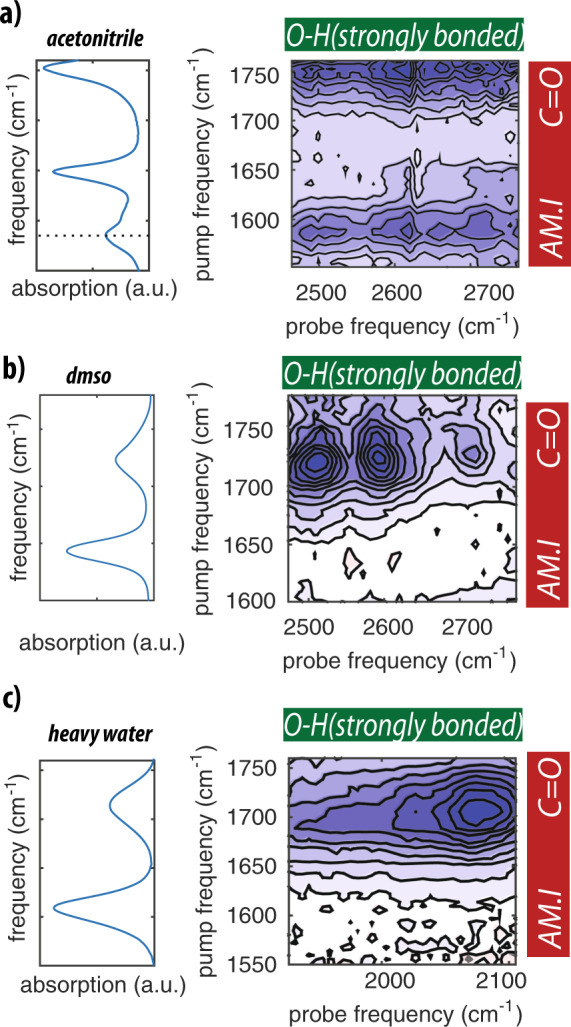
Intra-molecular hydrogen-bond between amide and carboxyl groups. Isotropic 2DIR spectra obtained by exciting amide I vibration, and probing the hydroxyl stretch vibration of *N*-acetylproline dissolved in acetonitrile (**a**), DMSO (**b**), and heavy water (**c**). All 2DIR spectra were collected at 0.5 ps. For the solution of heavy water the hydroxyl region is probed at 2050 cm^−1^.

The only moiety of *N*-acetylproline that can accept a strong hydrogen bond from the carboxyl O–H group is the amide group. To investigate the formation of an intramolecular hydrogen bond between the carboxyl O–H group and the C=O group of the amide group in more detail, we measured 2DIR spectra in which we tune the excitation pulses to the frequency range of the amide I vibration and the probing pulses to the frequency region low-frequency O–H stretch vibrations (Fig. 5). When *N*-acetylproline is dissolved in acetonitrile, we observe a strong cross-peak signal at an excitation frequency of 1585 cm^−1^ and probing frequencies near 2600 cm^−1^. We assign the excitation frequency of 1585 cm^−1^ to the amide I vibration of *N*-acetylproline. The frequency of this vibration is strongly red shifted compared to its usual value of  ~1650 cm^−1^ as a result of the formation of the strong intramolecular hydrogen bond with the carboxyl O–H group. This redshift is comparable to that of similar systems forming strong intra- and intermolecular hydrogen bonds^16–18^. The formation of this strong intramolecular hydrogen bond is further confirmed by the observation of an instantaneous cross-peak signal of the amide I vibration at 1585 cm^−1^ upon excitation of the carboxyl C=O vibration (see Supplementary Discussion and Supplementary Fig. 3). This signal demonstrates that the strong intramolecular hydrogen bond between the carboxyl O–H group and the amide group also leads to a coupling of the carbonyl vibration of the carboxyl group and the amide I vibration.

In Fig. 5b, c we show 2DIR spectra obtained by exciting in the frequency region of the amide I vibration and probing in the frequency region of the O–H stretch vibrations in DMSO solution (probing ~2600 cm^−1^) and in heavy water solution (probing ~2050 cm^−1^). The latter experiment was performed in heavy water to prevent absorption of the excitation pulses by the bending mode of H_2_O. For *N*-acetylproline dissolved in DMSO and in heavy water, we still observe a strong cross-peak signal between the C=O and the O–H vibrations of the carboxyl group, but no cross-peak signal of the amide I and the O–H stretch vibration. The absence of this cross-peak signal confirms that there is no strong intramolecular hydrogen bond formed between the amide and carboxyl groups when *N*-acetylproline is dissolved in DMSO solution or in water. This finding agrees with results of previous work^19–21^.

### Relative abundance of the different conformers of *N*-acetylproline

**Fig. 6:**
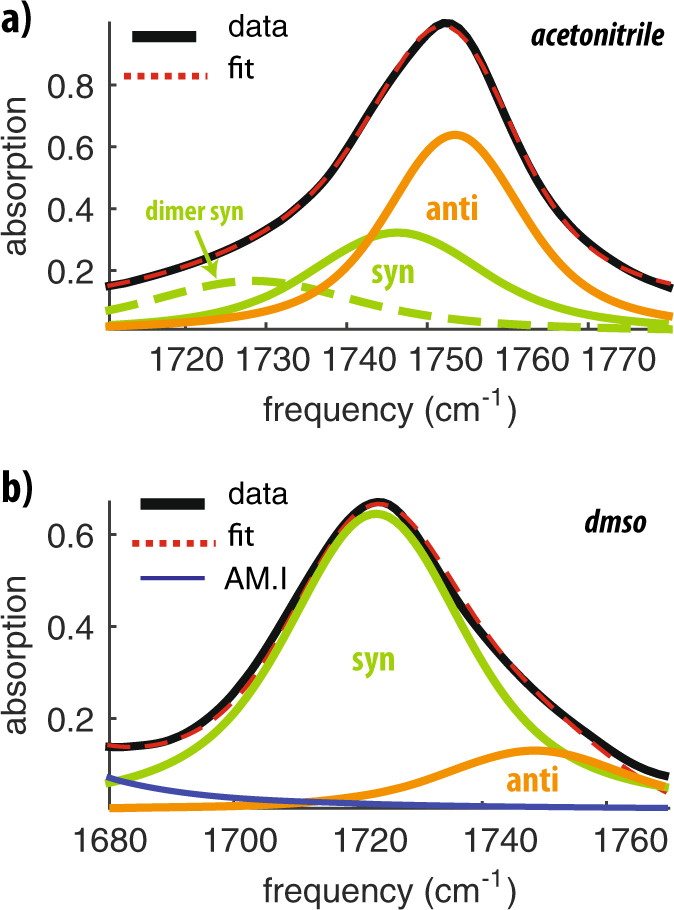
Abundance of syn- and anti- conformers in hydrophobic and hydrophilic environments. **a** Linear spectrum of the carboxyl C=O vibration of *N*-acetylproline in acetonitrile (0.08 M). We fit the spectrum with 3 Voigt-shaped bands to describe the response of the anti-conformer (*ν*_0_ = 1753 cm^−1^, *σ* = 6.1 cm^−1^, Γ = 6.3 cm^−1^), of the syn-conformer (*ν*_0_ = 1746 cm^−1^, *σ* = 8 cm^−1^, Γ = 8 cm^−1^), and of the syn-dimer (*ν*_0_ = 1728 cm^−1^, *σ* = 8, Γ = 12 cm^−1^). **b** Linear spectrum of the carboxyl C=O vibration of *N*-acetylproline in DMSO (0.4 M). We fit the spectrum with 2 Voigt-shaped bands to describe the response of the anti-conformer (*ν*_0_ = 1748 cm^−1^, *σ* = 11 cm^−1^, Γ = 11 cm^−1^) and of the syn-conformer (*ν*_0_ = 1723 cm^−1^, *σ* = 10 cm^−1^, Γ = 11 cm^−1^).

To quantify the relative abundances of the syn- and anti-conformers, we measure both linear infrared absorption spectra and degenerate 2DIR spectra of the carbonyl vibrations. In Fig. 6a we show the linear infrared absorption spectrum in the frequency region of the carboxyl C=O vibration for a solution of 0.08 M *N*-acetylproline in acetonitrile. We fit this spectrum with three Voigt-shaped bands, which account for both homogeneous and inhomogeneous broadening of each carbonyl absorption. Two of these bands are centered at the frequencies where we found the strongest syn and anti cross-peak signals (1747 and 1753 cm^−1^). The third band at 1728 cm^−1^ is assigned to the *N*-acetylproline dimer (see Supplementary Discussion and Supplementary Fig. 2). At 0.08 M the relative area of the anti-conformer band is 60 ± 10%. Figure 6b reports the spectrum of *N*-acetylproline in DMSO, which we fit with two Voigt-shaped bands centered at 1723 and 1749 cm^−1^. We find that in DMSO the relative area of the anti-conformer is only 15 ± 10%, which is thus much lower than in acetonitrile solution. We measure degenerate 2DIR spectra by tuning the excitation and probing pulses both to the frequency region of carbonyl vibrations (see Supplementary Discussion). We fit the diagonal slices of these spectra in the frequency region of the carbonyl vibration with two Voigt-shaped bands (Supplementary Fig. 4). With the band areas obtained from the linear and 2DIR spectra, we are able to determine the relative populations and cross-sections (see Supplementary Discussion and Supplementary Fig. 4). We find that the fractional area of the anti-conformer band in the 2DIR signal amounts to 65 ± 15% in acetonitrile solution and to 20 ± 10% in DMSO solution. These areas are similar to the areas found in the linear spectra, indicating that the carboxyl C=O vibration has a similar cross-section in the anti- and syn-conformations^9^. Unfortunately, we cannot analyze the data obtained for *N*-acetylproline in water in a similar manner, since the two carbonyl bands are broadened by strong interactions with the surrounding water molecules, and thus, overlap too strongly.

## Discussion

We find that the anti-conformer is much more abundant in acetonitrile solution than in DMSO and water solution. The relative fraction of the anti-conformer in acetonitrile solution is  ~60%, whereas in DMSO solution it is only  ~20%. This difference can be explained from the formation of a strong intramolecular hydrogen bond between the C=O group the amide in a trans conformation and the O–H group of the carboxyl group in an anti-conformation in acetonitrile solution. This hydrogen-bond formation is energetically sufficiently favorable to make the trans-anti conformational isomer the most abundant in this solution. In DMSO and water this intramolecular hydrogen bond is not formed and the syn-conformer of the carboxyl group prevails. This latter finding agrees with previous work on simple carboxylic acids like formic acid^9^ and acetic acid^22,23^. The syn-conformer is usually the more stable conformer, unless there exist specific interactions that strongly stabilize the anti-conformer.

An interesting question is why the intramolecular hydrogen bond would only be formed in acetonitrile solution and not in DMSO or water. We observe that the redshift of the O–H stretch vibration due to the formation of the amide-hydroxyl intramolecular hydrogen bond is similar to the redshift of the O–H stretch vibration observed upon dissolving *N*-acetylproline in DMSO or water. This finding indicates that the intramolecular hydrogen bond is of similar strength as the intermolecular hydrogen bonds to DMSO and water molecules, which makes it all the more surprising that there is no sign of the formation of an intramolecular hydrogen bond for *N*-acetylproline in DMSO and water. This observation indicates that other factors, such as a better solvation of the amide group and other parts of the molecule, also play a role, thereby making a configuration of *N*-acetylproline without intramolecular hydrogen bond more favorable in a polar solvent like DMSO or water.

The stabilization of the anti-conformer of the carboxyl group of amino-acids by the formation of an intramolecular hydrogen bond between the carboxyl O–H group and the amide C=O group may be a more general phenomenon. We found that the formation of this intramolecular hydrogen bond, and thus the relative abundance of the anti-conformer of the carboxyl group, strongly depends on the hydrophobicity of the surroundings of the amino acid. In a weakly hydrogen-bond accepting environment, as mimicked here by acetonitrile, such an intramolecular hydrogen bond is likely formed, leading to a strong increase of the relative abundance of the anti conformer. Hence, for C-terminal amino acids and aspartic and glutamic acid residues that are located in the hydrophobic micro-environment of a folded protein, the anti-conformer of the (side-chain) carboxyl group may be much more abundant than in a polar, aqueous environment, which may affect the secondary and tertiary structure of the protein.

In summary, we studied the molecular structure of the model acetylated amino acid *N*-acetylproline using linear infrared spectroscopy and two-dimensional infrared spectroscopy. *N*-acetylproline shows both amide and carboxyl conformational isomerization, and thus forms an interesting model system to study how the interaction of these groups influences the relative abundances of the different conformers of the carboxyl group of amino acids. When *N*-acetylproline is dissolved in acetonitrile, we observe a strong redshift of the O–H stretch vibrational frequency and a strong vibrational coupling of the amide vibration with both the C=O and O–H vibrations of the carboxyl group. These observations point at the formation of a strong intramolecular hydrogen bond between the amide C=O group and the carboxyl O–H group. This intramolecular hydrogen-bond formation is possible only in the trans-anti-configuration of *N*-acetylproline (Fig. 1). Due to the formation of a strong intramolecular hydrogen bond, the anti-conformer of the carboxyl group of *N*-acetylproline in acetonitrile has a relatively high abundance of 65 ± 15%, which is much higher than is observed for the carboxyl group of simple carboxylic acids like formic acid or acetic acid. If *N*-acetylproline is dissolved in a hydrogen-bond accepting solvent, like DMSO or water, the intramolecular hydrogen bond between the carboxyl and amide groups is not formed. In these latter solvents the relative abundance of the anti-conformer is only ~20%, similar to what has been observed for simple carboxylic acids.

## Methods

### Sample preparation

*N*-acetylproline (>95%, Enamine Ltd.) was dissolved in deuterated dimethyl sulfoxide (DMSO-d6, anhydrous, 99.8%, Sigma-Aldrich) or in acetonitrile (anhydrous, 99.8%, Sigma-Aldrich) or in ultrapure water or in heavy water (Cambridge Isotope Laboratories) to reach the desired concentration. For the infrared absorption measurements (Bruker Vertex 80v FTIR spectrometer) and the two-dimensional infrared experiments the solution was held between two calcium fluoride windows separated by a PTFE spacer of 10–50 μm thickness.

### Linear infrared spectroscopy

All linear absorption measurements were performed using a Bruker Vertex 80v FTIR spectrometer, equipped with a liquid-nitrogen-cooled mercury-cadmium telluride (MCT) detector. The spectra were recorded under a nitrogen atmosphere at a wavelength resolution of 3 cm^−1^. For every spectrum, 100 scans were averaged. In all measurements, a standard sample cell with a path length of 10–50 μm was used. The reported spectra were corrected for the absorption of the solvent background.

### 2DIR experiments

The details of our 2DIR setup can be found in the literature^24^. In brief, the pump and probe pulse were produced by two independently tunable optical parametric amplifiers pumped by the 800-nm output of a Ti:Sapphire regenerative amplifier. A Mach-Zehnder interferometer was used to generate the pump pulse pair (~100 fs, 5 μJ per pulse), which was subsequently focused in the sample to excite the carbonyl vibrations. This excitation induces transient absorption changes that are detected with a weak (0.35 μJ) single femtosecond probe pulse that is spatially overlapped with the pump pulse in the sample and delayed by a time *T*_*w*_. In all experiments the excitation pulses are centered at 1730 cm^−1^ with a bandwidth of 200 cm^−1^, in resonance with the carbonyl vibrations. The probe pulse was centered at 2650 or 3200 cm^−1^ to measure the response of the O–H stretch vibrations in dimethyl sulfoxide and water or in acetonitrile solutions. To obtain the degenerate spectrum, the probe pulse was centered at 1730 cm^−1^ to measure the response of C=O stretch vibrations. The polarization of probe pulse was rotated at 45° with respect to the pump by a half-wave plate. After the sample the probe was split and the parallel and perpendicular polarization components were dispersed by a monochromator and measured simultaneously by two arrays of a mercury-cadmium-telluride (MCT) detector, the third array was used to measure the reference pulse (split from the probe before the sample) to account for the pulse-to-pulse noise. The signal at each pixel was measured as a function of the delay time between the two pump pulses and then Fourier transformed to obtain transient absorption signal as a function of excitation frequency.

## Supplementary information

Supplementary Information

## Data Availability

The data that support the findings of this study are available from the corresponding author upon reasonable request.
