# Supplementary material for: Effect of intramolecular hydrogen-bond formation on the molecular conformation of amino acids
Source: Commun Chem. 2020 Jun 30;3:84. doi: 10.1038/s42004-020-0329-7 (PMC9814578; doi:10.1038/s42004-020-0329-7)
Supplement: Supplementary file 1 — Supplementary Information [file 42004_2020_329_MOESM1_ESM.pdf]

Supplementary Information
Effect of intramolecular hydrogen-bond formation on the molecular conformation of amino-acids

Giulia Giubertoni[1,†],Oleksandr O. Sofronov[1,†], Huib J. Bakker[1,*]

[1] *AMOLF, Science Park 104, 1098 XG Amsterdam, The Netherlands*

[†] *These authors equally contributed to the work*

Supplementary Discussion

Angle between the carbonyl and the hydroxyl groups

In our 2DIR setup, signals in parallel and perpendicular polarizations are acquired simultaneously. For each band we calculate the anisotropy $R = \frac{\Delta\alpha_{par} - \Delta\alpha_{per}}{\Delta\alpha_{par} + 2\Delta\alpha_{per}}$, where $\Delta\alpha_{par}$ and $\Delta\alpha_{per}$ are the transient absorption changes (cross-peak signals) measured in parallel and in perpendicular polarization configuration, respectively. The anisotropy represents the relative orientation of the hydroxyl transition dipole moment with respect to the carbonyl transition dipole moment, and can be used to calculate the angle between the carbonyl and the hydroxyl groups following $\theta = \arccos\sqrt{\frac{5R_0+1}{3}}$.

Dimer formation of N-acetylproline in acetonitrile

Supplementary Figure 1 shows that the increase of the concentration of N-acetylproline leads to the rise of an additional amide I absorption band at 1606 cm^{-1} and an additional carboxyl C=O stretch absorption band at 1728 cm^{-1}. The additional carboxyl C=O stretch is also observed in the 2DIR spectrum, as illustrated in Supplementary Figure 2a. The rise of these bands indicates the formation of dimers of N-acetylproline. At a concentration of 0.2 M, a second C=O/O-H cross-peak appears in the 2DIR spectrum at a relatively low excitation frequency of $\sim$1728 cm^{-1}. This signal is stronger for the perpendicular polarization configuration than for the parallel polarization configuration, which indicates that this C=O vibration corresponds to a dimer of *syn*-conformers.

Excitation of the amide vibration of the N-acetylproline dimer at 1605 cm^{-1} gives rise to a cross-peak signal with strongly hydrogen-bonded hydroxyl vibrations (Supplementary Figure 2b). This observation shows that the dimer formation involves the interaction between the carboxyl group of one N-acetylproline molecule and the amide group of another N-acetylproline molecule. Since in this dimer the hydroxyl group is strongly hydrogen bonded to the amide group of the second molecule, both the carbonyl and hydroxyl vibrations shift to lower frequencies.

Cross-peak between amide I and carbonyl vibrations of N-acetylproline

To study the interaction between the amide and the carboxyl groups in more detail, we also measured 2DIR spectra in the C=O/amide I cross-peak region. In Supplementary Figure 3a we observe that excitation of the carboxyl C=O vibration at 1750 cm^{-1} leads to cross-peak signatures of two amide I modes at 1650 and 1585 cm^{-1}. To understand the origin of these cross-peaks, we resolve their dynamics. In this analysis we average over the pump frequency region between 1700 and 1770 cm^{-1} to obtain 2DIR signals as a function of probe frequency for time delays between 0.3 and 10 ps (Supplementary Figure 3b). We then fit the resulting transient absorption spectra with a cascade kinetic model (A$\rightarrow$B$\rightarrow$C). We observe that the spectral signature of state A contains a cross-peak signal at a probe frequency of 1583 cm^{-1}, showing that this cross-peak is instantaneous. This means that the carboxyl C=O and the amide I vibrations are anharmonically coupled, as a result of the strong hydrogen bond that is formed between the carboxyl O-H group and the amide C=O group. The signature of the cross-peak at ν_{probe} =1650 cm^{-1} is not present in the initial spectral component A, and is part of component B that rises with a time constant of 0.6 ps (Sup-

plementary Figure 3c). This finding suggests that the cross-peak at ν_{probe} =1650 cm^{-1} results from intramolecular energy transfer.

Determination of the relative areas of syn- and anti- conformers of 2DIR diagonal slices

The relative concentrations of the conformers cannot be directly determined from the areas of the corresponding bands in the linear infrared absorption spectrum as the cross-sections of the carbonyl vibrations of the two conformers are not known. However, we can make use of the fact that the linear infrared absorption spectrum scales with σ (where σ is the absorption cross-section), while the 2DIR spectrum scales with σ^2, thus providing an additional relation between the measured signals, cross-sections and concentrations.

Supplementary Figure 4 shows 2DIR diagonal slices of the negative absorption change peaks of N-acetylproline in acetonitrile and DMSO as a function of pump frequency. We fit the 2DIR signal by using two Voigt-shaped bands, which represent the carbonyl stretching vibrations of *syn*- and *anti*-conformers (colored in green and orange, respectively). We find that in acetonitrile the area of the *anti*-conformer band amounts to 65$\pm$15$\%$ of the total 2DIR signal, which is similar to the relative fraction derived from the linear spectra. This finding implies that the cross-sections of the carboxyl C=O vibrations of the *anti*- and *syn*-conformers are similar. We also fit the diagonal slice of the 2DIR spectrum of N-acetylproline in DMSO, and we find that the relative area of the *anti*-conformer band (20$\pm$10$\%$) is also similar to that derived from the linear spectrum, showing that also in DMSO the carboxyl C=O vibrations of the two conformers have similar cross-sections.

Supplementary Figures

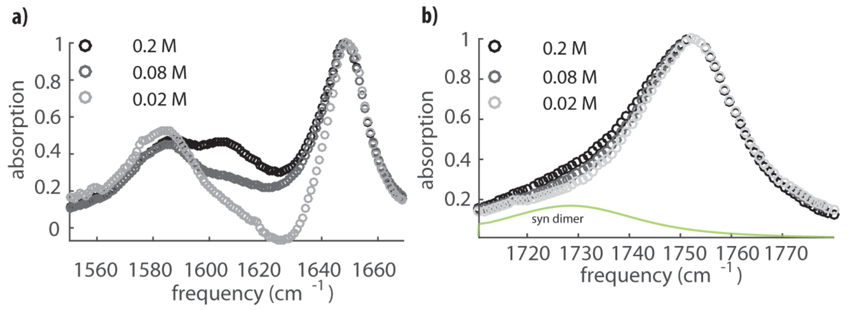

Supplementary Figure 1: Normalized linear spectra of N-acetylproline at concentration of 0.02, 0.08, and 0.2 M in the amide I (a) and carboxyl C=O stretch (b) vibrational regions. In a) we observe an increase of absorption at 1606 cm^{-1} with increasing N-acetylproline concentration, while in b) we observe an increase of absorption around 1725 cm^{-1} with increasing concentration.

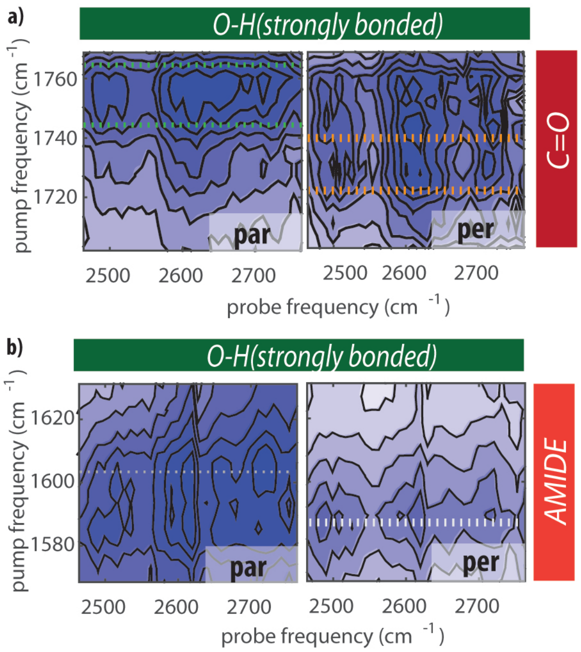

Supplementary Figure 2: 2DIR spectra of N-Acetylproline in acetonitrile at a concentration of 0.2 M, obtained by exciting in the frequency region of the carboxyl C=O vibrations (a) and in the frequency region of the amide I vibrations (b). In both cases, we probe in the frequency region of the OH stretch vibrations around 2600 cm^{-1}. The 2DIR spectra are recorded in parallel (left panel) and perpendicular (right panel) polarization configurations. In a) the green bar shows the cross-peak between the carbonyl and the OH vibration of the *anti*-conformer, while the orange bar shows the cross-peak the carbonyl and the OH vibration of the dimer. In b) the white lines show the central frequencies of the amide I vibration in the dimer (left) and in the trans-*anti* conformer (right) of N-acetylproline.

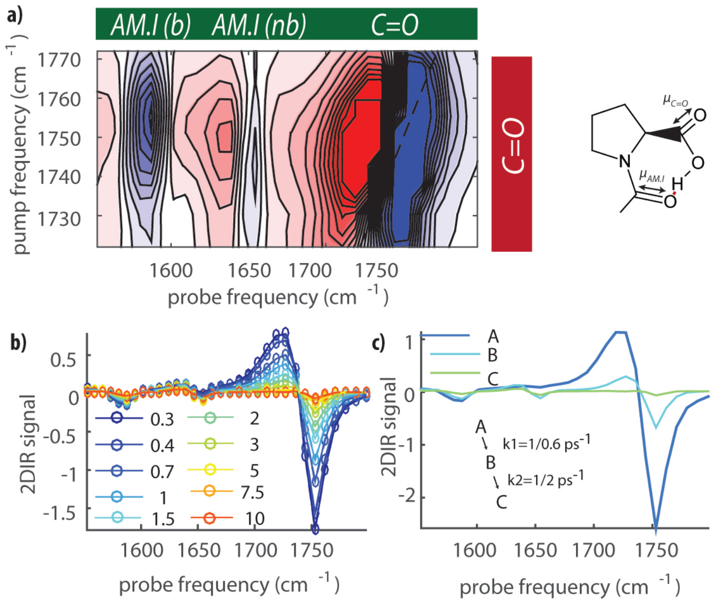

Supplementary Figure 3: a) 2DIR spectrum of N-acetylproline in acetonitrile at a concentration of 0.08 M measured by exciting in the frequency region of the carboxyl C=O vibrations and by probing in the frequency region of the amide I and carboxyl C=O vibrations at a waiting time T_w of 0.5 ps. b) 2DIR probe slices obtained by averaging over the pump frequency region 1700-1770 cm^{-1} at different waiting times. c) The spectral signatures and interconversion time constants of the three states used to fit the spectral dynamics.

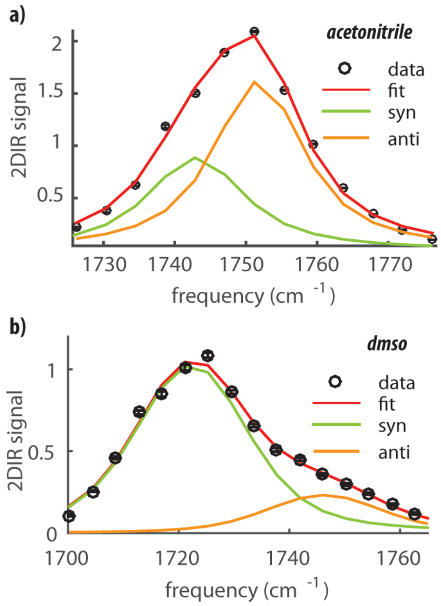

Supplementary Figure 4: a) Diagonal slice of the 2DIR spectrum of N-acetylproline in acetonitrile (0.08 M). We fit the spectra with 2 Voigt-shaped bands to describe the response of the *anti*-conformer ($\nu_0 = 1752\ cm^{-1}$, $\sigma = 8.2\ cm^{-1}$, $\Gamma = 6.5\ cm^{-1}$), and of the *syn*-conformer ($\nu_0 = 1743$ cm^{-1}, $\sigma = 9\ cm^{-1}$, $\Gamma = 5\ cm^{-1}$). b) Diagonal slice of the 2DIR spectrum of N-acetylproline in DMSO (0.4 M). We fit the spectra with 2 Voigt-shaped bands to describe the response of the *anti*-conformer ($\nu_0 = 1722\ cm^{-1}$, $\sigma = 9\ cm^{-1}$, $\Gamma = 6\ cm^{-1}$), and of the *syn*-conformer ($\nu_0 = 1746$ cm^{-1}, $\sigma = 9\ cm^{-1}$, $\Gamma = 7\ cm^{-1}$).
